# Cardiovascular Risk and Endothelial Dysfunction in Primary Sjogren Syndrome Is Related to the Disease Activity

**DOI:** 10.3390/nu13062072

**Published:** 2021-06-17

**Authors:** Anna Łuczak, Rafał Małecki, Michał Kulus, Marta Madej, Ewa Szahidewicz-Krupska, Adrian Doroszko

**Affiliations:** 1 Department of Rheumatology, Wroclaw Medical University, Borowska 213, 50-556 Wroclaw, Poland; ajluczak@wp.pl (A.Ł.); marta.madej@umed.wroc.pl (M.M.); 2 Department of Angiology, Hypertension and Diabetology, Wroclaw Medical University, Borowska 213, 50-556 Wroclaw, Poland; rafal.malecki@umed.wroc.pl; 3 Department of Histology and Embryology, Wroclaw Medical University, Chalubinskiego 6a, 50-367 Wroclaw, Poland; michal.kulus@umed.wroc.pl; 4 Department of Internal Medicine, Occupational Diseases, Hypertension and Clinical Oncology, Wroclaw Medical University, Borowska 213, 50-556 Wroclaw, Poland; ewa.szahidewicz-krupska@umed.wroc.pl

**Keywords:** primary Sjogren syndrome, endothelial dysfunction, ADMA, flow-mediated dilatation

## Abstract

The aim of our study was to evaluate if endothelial-dysfunction (ED) occurs in patients with primary Sjogren syndrome (pSS) and whether it is associated with the disease characteristics and activity. A total of 46 patients with pSS and 30 controls, without known cardiovascular disease, were enrolled in this study. A flow-mediated-dilation (FMD) of the brachial artery, plasma concentrations of the nitric oxide (NO) metabolic pathway (ADMA, L-arginine, SDMA, cGMP), and markers of endothelial inflammatory function (PAI-1, sE-selectin) and angiogenesis (angiostatin, VEGF) were analyzed. The FMD was significantly lower in pSS patients (7.56 ± 3.08 vs. 10.91 ± 1.02%, *p* = 0.043) and positively correlated with the Ro/SS-A-antibodies (*r* = 0.34, *p* = 0.03), pulmonary involvement (*r* = 0.52, *p* = 0.001) and inversely with ADMA (*r* = −0.35, *p* = 0.04). Plasma ADMA, L-arginine and angiostatin levels were significantly higher in pSS patients (0.39 ± 0.08 vs. 0.36 ± 0.06 µmol/L, *p* = 0.05; 29.07 ± 6.7 vs. 25.4 ± 5.23 µmol/L, *p* = 0.01; 152.25 ± 60.99 vs. 120.07 ± 38.7 pg/mL, *p* = 0.0, respectively). ADMA was associated with ESSDAI (*r* = 0.33, *p* = 0.02), SCORE (*r* = 0.57, *p* = 0.00003) and focus score (*r* = 0.38, *p* = 0.04). In the multiple regression analysis, the ESSDAI was significantly and independently associated with plasma ADMA levels (*β* = 0.24, *p* = 0.04). Moreover, plasma cGMP concentrations were negatively correlated with the disease duration (*r* = −0.31, *p* = 0.03). Endothelial function is impaired in patients with pSS and associated with the measures of disease activity, which supports the key-role of inflammation in developing and maintaining accelerated atherosclerosis.

## 1. Introduction

Cardiovascular disease (CVD), due to accelerated atherogenesis, has been recognized as an important morbidity and mortality cause in rheumatic autoimmune diseases. This excess CV burden is partly mediated by chronic systemic inflammation along with the increased prevalence of conventional risk factors. Inflammation can accelerate the atherogenesis via inflammatory cell infiltration into the vascular wall and endothelial dysfunction (ED). Moreover, inflammation may act synergistically with existing traditional risk factors or attenuate the effects of factors protective for CVD [1,2].

Up to date, some evidence suggests that the vascular endothelium may be a key interface between inflammation and atherogenesis and its altered function precedes the development of morphological changes. Thus, endothelial dysfunction is currently considered the earliest stage of induction and maintenance of atherosclerosis [1,3]. Furthermore, many studies have shown that endothelial function improves with risk reduction therapies or during treatment with anti-rheumatic drugs [4,5].

Since endothelial dysfunction is a systemic condition, the impaired ultrasound assessment of brachial artery flow-mediated dilation (FMD) is predictive of ED in other vascular beds, including coronary vessels. Therefore, in recent years the FMD measurement has emerged as a valuable non-invasive method to study endothelial function as a marker of subclinical atherosclerosis, as well as a prognostic tool in predicting development and clinical outcome of CVD [3,6]. Nevertheless, apart from vascular tone regulation, endothelium plays multiple roles in the maintenance of vascular wall homeostasis including control of inflammation and smooth muscle cell proliferation, regulation of platelet adhesion and aggregation, and modulation of thrombosis and fibrinolysis. All these actions are exerted through synthesis and expression of numerous vasoactive molecules [7].

Recent epidemiologic data indicates an increase in cardiovascular risk in patients with primary Sjogren syndrome (pSS). Particularly, pSS has been associated with almost 1.5-fold increased risk of both cardiac and cerebrovascular events in comparison to the general population. However, the mechanisms underlying elevated CV risk in pSS remain poorly understood [8,9]. The few previously conducted studies examining subclinical atherosclerosis and endothelial damage have shown conflicting results [10,11,12,13,14,15].

To date, however, no study has examined the ED in a comprehensive manner in pSS patients. Therefore, the aim of this study was to evaluate if endothelial dysfunction occurs in patients with primary Sjogren syndrome and whether it is associated with the disease characteristics and activity.

## 2. Materials and Methods

### 2.1. Study Population

**Figure 1:**
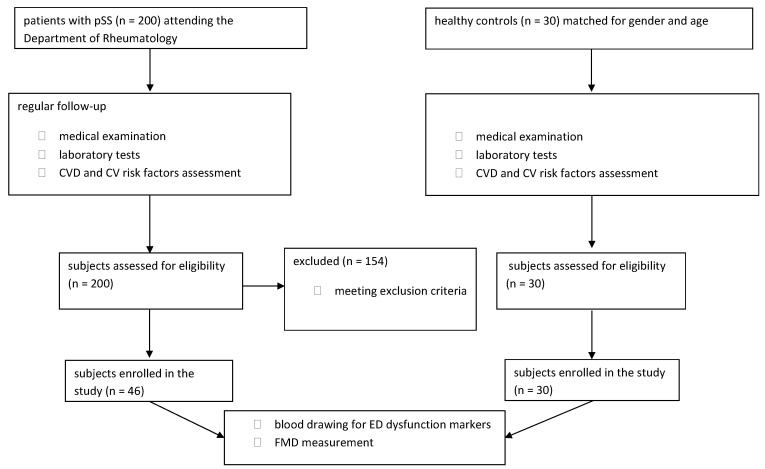
A flow chart presenting the subjects’ recruitment and analyses performed in this study. Abbreviations: CV—cardiovascular; CVD—cardiovascular disease; ED—endothelial dysfunction; FMD—flow mediated dilation; pSS—primary Sjogren syndrome.

A total of 200 patients with pSS clinically diagnosed strictly according to the 2016 ACR (American College of Rheumatology)/EULAR (European League Against Rheumatism) classification criteria at the Department of Rheumatology at Wroclaw Medical University Hospital were included in this study (Figure 1) [16]. The patients were undergoing follow-up on a regular basis in the clinic according to a standard protocol every 6–12 months. Demographic and clinical data including CV risk factors were collected by a questionnaire, and physical examination including a structured interview. Blood samples were collected for subsequent biochemical and immunological analyses performed as a part of routine care. Disease activity was measured by means of EULAR Sjogren’s Syndrome Disease Activity Index (ESSDAI) and severity of patients’ symptoms was assessed by EULAR Sjogren’s Syndrome Patient Reported Index (ESSPRI) [17,18]. CVD risk stratification was evaluated using the Systematic Coronary Risk Evaluation (SCORE) algorithm [19]. Physical activity was defined as 5 or more times a week of at least 30 min of moderately intense physical exercise. The study protocol had been accepted by the local Bioethics Committee (KB 390/2015) and written informed consent was obtained from each participant of this study.

### 2.2. Exclusion Criteria

The exclusion criteria were designed to include patients without conventional cardiovascular risk factors and without factors influencing endothelial function, in order to be able to clearly assess the impact of pSS on the phenotype of endothelial function and the cardiovascular risk profile.

Exclusion criteria applied to both groups and included: pregnancy, age >65 years, renal dysfunction (serum creatinine level >3 mg/dL (>264 mmol/L), estimated glomerular filtration rate (eGFR) < 30 mL/minute), malignancies, infectious diseases, other systemic autoimmune diseases, endocrine disorders (including diabetes mellitus and thyroid disorders), obesity (as assessed by the body mass index (BMI) ≥ 30 kg/m^2^), smoking history, hypertension (classified using the WHO definitions), dyslipidemia (defined according to the 2019 ESC/EAS guidelines) [20], personal history of CVD (including coronary artery disease, ischemic cerebrovascular disease, peripheral vascular disease and major ischemic vascular events and/or revascularization procedures) and family history of CVD (defined as a cardiovascular episode occurring at age <55 for men or 65 for women in first-degree relatives, respectively). Patients’ further exclusion criteria were ongoing/or previous antiplatelet agents and/or nonsteroidal anti-inflammatory drugs (NSAIDs) and/or corticosteroids therapy and disease duration shorter than 1 year following the diagnosis. As a final result, 46 subjects (43 females, 3 males) and 30 healthy controls matched for gender, age and other anthropometric and sociodemographic characteristics were recruited.

### 2.3. Laboratory Tests

#### 2.3.1. Markers of Endothelial Activation

Venous blood samples were collected from each participant under fasting conditions. Besides biochemical and immunological analyses performed as a part of routine care using standardized laboratory tests, plasminogen activator inhibitor-1 (PAI-1), E-selectin, angiostatin, cyclic guanosine monophosphate (cGMP) and vascular endothelial growth factor (VEGF) were measured. Plasma concentrations of cGMP and PAI-1 and serum concentrations of VEGF and E-selectin were evaluated using commercial ELISA kits (R&D Systems Europe Ltd., London, UK). Serum concentrations of angiostatin were determined using ELISA kit ELH-Angiostatin (RayBiotech, Inc., Norcross, GA, USA). All tests were conducted according to the manufacturer’s instructions. The coefficient of variation (CV)—intra-assay percentage CV and inter-assay were respectively: 6.3% and 7.1% for PAI-1; 6.7% and 8.2% for E-Selectin; 5.6% and 7.2% for VEGF; 7.1% and 8.9% for cGMP; 9.2% and 10.3% for angiostatin. 

#### 2.3.2. Markers of the Nitric-Oxide-Metabolic-Pathway

Plasma levels of L-arginine and its methyl-derivatives asymmetrical dimethylarginine (ADMA) and symmetrical dimethylarginine (SMDA) were measured by high-performance liquid chromatography (HPLC). The samples of standard and plasma extracted a solid-phase-extraction cartridge with SCX50 columns (Varian Inc., Palo Alto, CA, USA). The eluates were derivatized with *o*-phthaldi-aldehyde (OPA) which was followed by separation via isocratic reversed-phase chromatography on a Symmetry C18 column (150  ×  4.6  mm, 5  μm particle size; Waters Corp., Milford, MA, USA). The potassium-phosphate buffer (50  mM, pH  6.6) containing 12% acetonitrile at a flow rate of 1.1  mL/min was used as the mobile phase. Fluorescence detection was conducted at excitation 340  nm and emission 450  nm wavelengths. The test was performed on a computer controlled by Star Chromatography Workstation software (version 6.3); the device was made by Varian (New York, NY, USA).

#### 2.3.3. Measurement of Vascular Function

Ultrasound assessment of the brachial artery FMD in response to reactive hyperemia was performed according to the method published by Celermajer et al. using the ALOKA model alpha 10 duplex ultrasound machine (model ALOKA GmbH, Willich, Germany) with a 7–14-MHz linear array transducer [21]. In order to improve the accuracy of FMD measurement, the ALOKA probe holder MP-AH 0001 was used to immobilize the arm/forearm.

### 2.4. Statistical Analysis

Data is expressed as the mean ± SEM. The differences between two continuous variables were assessed using a Mann–Whitney-U-test or a Student’s t-test, which was followed by a Shapiro-Wilk test and Levene’s test, as appropriate. For comparison of more than two groups, an ANOVA followed by Tukey’s test or a Kruskal–Wallis test (for non-parametric statistics) was conducted. Correlations were assessed with the univariate (Pearson Moment or Spearman’s test as appropriate) and multivariate linear regression analyses. All calculations were performed using the Statistica 10.0 software (StatSoft^®^ Krakow, Poland).

## 3. Results

### 3.1. Characteristics of the Study Group

The baseline disease characteristics of the subjects and selected demographic, laboratory and cardiovascular characteristics of all subjects are presented in Table 1 and Table 2, respectively.

### 3.2. Endothelial Function in pSS

**Figure 2:**
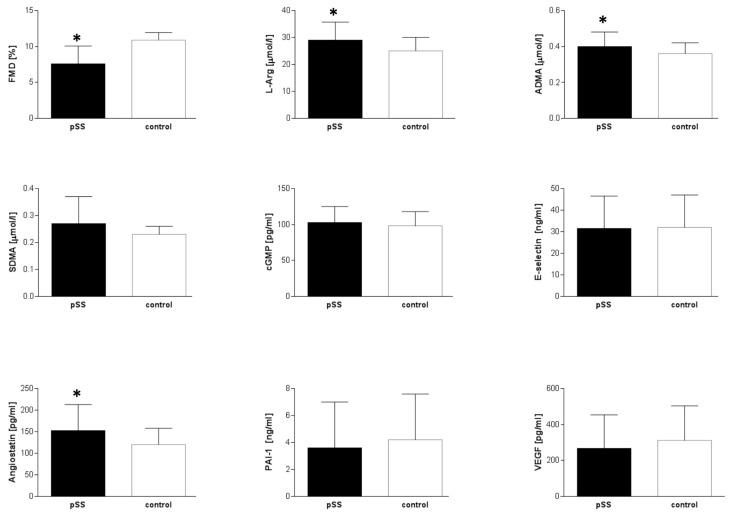
Endothelial function profile. Abbreviations: ADMA—asymmetric dimethylarginine, cGMP—cyclic guanosine monophosphate, FMD—flow mediated dilation, PAI-1—plasminogen activator inhibitor-1, SDMA—symmetric dimethylarginine, VEGF—vascular endothelial growth factor, * *p* < 0.05 vs. control.

Patients with pSS had significantly elevated plasma ADMA, L-arginine and angiostatin levels (0.39 ± 0.08 vs. 0.36 ± 0.06 µmol/L, *p* = 0.05; 29.07 ± 6.7 vs. 25.4 ± 5.23 µmol/L, *p* = 0.01; 152.25 ± 60.99 vs. 120.07 ± 38.77 pg/mL, *p* = 0.01, respectively) and impaired FMD (7.56 ± 3.08 vs. 10.91 ± 1.02%, *p* = 0.043) compared with controls (Table 3). Analyses of the relationships between FMD and serum endothelial biomarkers showed negative correlation between the FMD and ADMA (*r* = −0.35, *p* = 0.05), L-arginine (*r* = −0.35, *p* = 0.03) and VEGF levels (*r* = −0.44, *p* = 0.006) (Figure 2).

The correlations of endothelial dysfunction with disease-specific features and activity in pSS patients are presented in Table 3.

## 4. Discussion

This is the first study to thoroughly evaluate endothelial function in subjects with primary Sjogren syndrome without clinically evident cardiovascular disease or presence of concomitant cardiovascular risk factors. Moreover, to the best of our knowledge, this is the first study to show the association of endothelial dysfunction with disease activity and to explain partially the molecular mechanisms linking endothelial dysfunction with clinical features of the disease. Interestingly, the study by Cinoku et al. [22] gives a wider view on the complex nature of atherosclerosis in general population, supporting the recent findings that autoimmune mechanisms seem to be involved in the pathophysiology of atherosclerosis. The authors postulate that atherosclerosis should be considered an autoimmune disease and provide us with substantial evidence to back up this thesis, and their considerations go far beyond the endothelial cell. They also point at other immune cell populations which display a significant and prominent role in atherogenesis, and show the true pathogenic potential of oxidized form of low-density lipoprotein (ox-LDL) as initial triggers of atherosclerotic disease. Recent epidemiologic data indicates an increase in cardiovascular risk in patients with pSS. Thus, to find molecular mechanisms underlying this phenomenon, our study thoroughly evaluates endothelial function in pSS patients without clinically evident cardiovascular disease or presence of concomitant conventional cardiovascular risk factors. Knowing that inflammation can accelerate atherogenesis, we investigated whether chronic systemic inflammation can alter endothelial function and searched for an association between endothelial functional phenotype and disease activity.

In the present study, the FMD was significantly impaired in the pSS patients compared with the group of healthy controls matched for age and sex. Moreover, FMD was impaired despite a short disease duration and a moderate disease activity, suggesting that the disease itself contributes to endothelial dysfunction and development of accelerated atherosclerosis. The association of FMD with the presence of circulating anti-Ro/SSA antibodies, a well-established marker of the disease strongly related with systemic manifestations and increased severity, provided further rationale to support this hypothesis. Indeed, immune system dysregulation plays a pivotal role in the pathogenesis of precocious atherosclerosis in pSS patients. The autoantibodies can induce production of type I interferons (IFN), which as recent evidence indicates could play a prominent role in endothelial cell damage and contribute to the development of cardiovascular disease [23,24]. Corroborating the animal and some in vitro data, type-I IFN signatures or high serum IFN-α are associated with accelerated endothelial cell apoptosis, impaired endothelial progenitor cell (EPC) function, reduced flow-mediated dilation, increased foam cell formation, enhanced pro-inflammatory leukocyte recruitment, platelet activation with subsequent thrombosis and elevated endothelial activation markers in systemic lupus erythematosus (SLE), antiphospholipid syndrome (APS) and rheumatoid arthritis (RA) [25,26,27]. Moreover, animal and human studies suggest that IFN-α causes the depletion of tetrahydrobiopterin (BH_4_) via oxidation, serving as a potential mediator of endothelial NO synthase (eNOS) uncoupling and oxidative stress. Therefore, this mechanism could at least partially explain the elevated ADMA levels observed in rheumatic autoimmune diseases, since ROS have been demonstrated to increase the activity of arginine-methylating enzymes, protein-arginine methyl transferases (PRMTs), and to inhibit that of ADMA-degrading enzyme dimethylarginine dimethyl-amino-hydrolase (DDAH) [28].

In this study, the plasma ADMA levels were significantly higher in pSS patients than controls and were negatively correlated with FMD, supporting the hypothesis that endothelial dysfunction is related to altered NO bioavailability [1]. The high ADMA concentration was independent of all traditional CV risk factors; however, it was simultaneously associated with global cardiovascular risk as assessed by the SCORE index. In addition, plasma ADMA levels were significantly associated with measures of disease activity (ESSDAI and focus score). These results strongly indicate the association between chronic inflammation and increased ADMA generation. ADMA synthesis and degradation are regulated by inflammatory activity and, on the other hand, ADMA contributes to the development of inflammation-related endothelial dysfunction via increase in tumor necrosis factor-alpha (TNF-α) concentrations in human-endothelial-cells, decreased NO production by eNOS inhibition and enhanced oxidative stress by eNOS uncoupling [28,29].

Persistent inflammation and angiogenesis are known to be interdependent. In the RA, SLE and ankylosing spondylitis, serum VEGF levels reflect disease activity and internal organ involvement [30,31]. In this study, there was no significant difference in the plasma VEGF levels among the groups which is probably due to a moderate disease activity. Similar findings were observed in the RA—patients with a moderate disease activity were characterized by a similar VEGF production as in healthy controls [32]. At the same time, VEGF correlated with the presence of hypo-complementemia, a serological sign in pSS of ongoing inflammation and systemic expression and predictor of the adverse outcomes (lymphoma development and death), which also supports the role of VEGF as disease activity marker in pSS. On the other hand, similar blood levels of VEGF in both groups may be a consequence of the treatment effects on factor serum levels or enhanced level of angiostatin, which effectively blocks the VEGF pathway [33].

**Figure 3:**
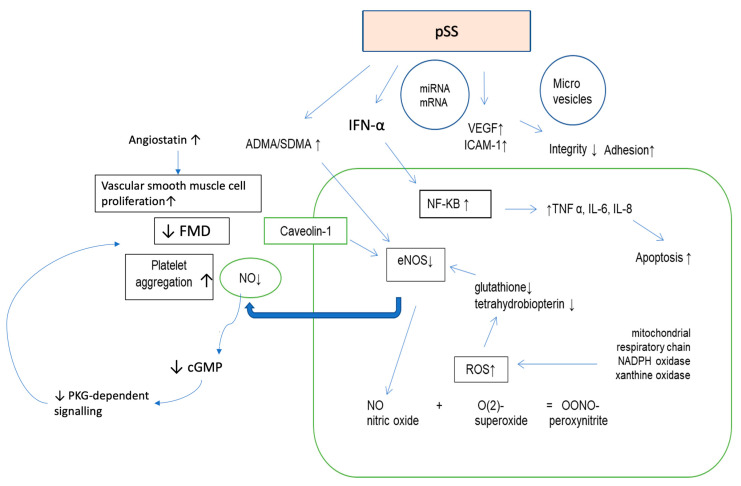
Changes in the profile of endothelial function in the primary Sjogren syndrome. Abbreviations: VEGF: vascular endothelial growth factor; ICAM-1: intercellular adhesion molecule 1; IFN-α: interferone alpha; NF-κB: nuclear factor kappa-light-chain-enhancer of activated B cells; TNFα: tumor necrosis factor alpha; IL-6: interleukin 6; IL-8: interleukin 8; SDMA: symmetric dimethylarginine; ADMA: asymmetric dimethylarginine; ROS: reactive oxygen species; eNOS: endothelial nitric oxide synthase; ONOO-: peroxynitrite; NO: nitric oxide; FMD—flow-mediated dilation; cGMP—cyclic guanyl monophosphate; O(2)-: superoxide anion; PKG—protein kinase G; pSS: primary Sjogren syndrome, ↑: increased; ↓: decreased.

Increased angiostatin levels and the inverse association between VEGF and FMD observed in this study indicate that impaired angiogenesis could be a result of impaired endothelial function, which was demonstrated in animal models [34]. Inflammation might interfere with the function of endothelium through the proinflammatory cytokines which are capable of modifying the vascular release of nitric oxide. Reduced NO generation and/or bioavailability along with increased reactive oxygen species (ROS) production, downregulate VEGF expression and promote angiostatin production [35]. The up-regulation of the anti-angiogenic mediator could, on the one hand, contribute to ischemic damage, since angiostatin acts as a negative regulator of endothelial-dependent vasodilation by uncoupling eNOS activity, but on the other hand it might counterbalance the inflammation-induced angiogenesis [36]. Recent data shows that angiostatin inhibits the nuclear factor kappa-light-chain-enhancer of activated B cells (NF-κB) activation via TNF-α and interleukin-1 beta (IL-1β), mediators that develop and maintain inflammatory response through subsequent expression of chemotactic factors and adhesion molecules in endothelial cells. Therefore, angiostatin inhibits both endothelial activation and dysfunction as it directly inhibits neutrophils and monocyte activation, migration, and neutrophil-mediated angiogenesis [37]. In the present study, angiostatin levels inversely correlated with the anti-Ro/SS-A antibodies and hypergammaglobulinemia; therefore we postulate that angiostatin may serve as an anti-inflammatory factor in pSS (Figure 3). However, the role of angiogenic and anti-angiogenic factors in pSS has not yet been investigated and requires further study.

The absence of association between disease duration and subclinical atherosclerosis (as assessed by FMD and serum biomarker levels) is surprising, as many studies in rheumatoid arthritis and/or lupus erythematosus have shown an association between longer disease duration and higher cardiovascular risk [38,39]. This finding might be explained by the heterogeneous characteristics of pSS, as well as its variable course.

Interestingly, however, an inverse correlation between disease duration and cGMP was found. The cGMP is a cardioprotective second messenger and alterations in the cGMP signaling cascade have been implicated in the pathogenesis of several cardiovascular disorders. cGMP regulates blood pressure by stimulating vasodilation and natriuresis and by inhibiting the renin–angiotensin–aldosterone system (RAAS). Decrease in cGMP signaling in the blood vessels or in the kidney contributes to the development and maintenance of hypertension. Therefore, it may be postulated that this association could partially explain the higher prevalence of hypertension in patients with greater disease duration [40,41].

It is noteworthy that from the cohort of 200 subjects only a small number of pSS patients was finally included into the study after applying the exclusion criteria. Therefore, these results are in keeping with previously reported data that patients with pSS have increased prevalence of CV risk factors—diabetes mellitus, hyper-lipidaemia and hypertension [42,43]. Moreover, in the studied pSS group blood pressure was higher than in controls, although both groups were normotensive according to study protocol and the values were within the range of optimal blood pressure, according to the European Society of Hypertension (ESH). Some evidence indicates that endothelial dysfunction, resulting from impaired activity of the NO-dependent metabolic pathways, is considered to have a causative role in hypertension development, since decreased NO bioavailability promotes increase in peripheral arterial resistance, reduces renal ability to excrete sodium and increases angiotensin II (Ang II) formation via synthesis of angiotensin-converting enzyme (ACE) and Ang II type 1 receptors. Furthermore, ED in addition to impairment of FMD, may cause smooth muscle proliferation and vascular fibrosis, leading to arterial stiffness, which precedes development of high blood pressure. On the other hand, there is still the question as to whether endothelial dysfunction is a cause or consequence of hypertension and current data support a complex and potentially bidirectional relationship [44,45]. Our data, however, is in support of endothelial dysfunction preceding hypertension, but in order to verify this hypothesis a larger population of subjects should be investigated.

However, several other factors may also contribute to elevated CV risk. pSS affects predominantly post-menopausal women with a peak age of onset in the fourth and fifth decades of life, when the protective effect of female hormones is fainting. Immunosuppressive therapy, especially with gluco-corticosteroids, independently increases the CV risk through an array of side effects that often lead to development of metabolic syndrome. Similarly, a sedentary lifestyle associated with a low quality of life due to fatigue and depression, commonly observed in patients with pSS, worsens their metabolic profile [43]. However, questions as to if, how and to what extent these concomitant factors account for a higher incidence and prevalence of traditional risk factors in pSS represents a research area yet to be explored.

A negative relationship between FMD and ESSPRI found in this study supports the use of the ESSPRI in daily clinical practice. This very simple index can be self-administered in two minutes and scored in less than one minute. Fairly limited evidence has shown that in addition to the ESSDAI, the ESSPRI could at least partly reflect inflammatory disease activity in patients with pSS [22]. Furthermore, along with more prominent ESSDAI, it has emerged as a sensitive indicator to measure change in disease activity after therapeutic intervention [46]. Therefore, this simple instrument, along with changes in its values during follow-ups, could offer additional information for preliminary assessment of potential CV risk.

These findings are of a particular interest from a clinical point of view. Our study shows that patients with pSS without an overt cardiovascular disease and major atherosclerotic risk factors already have altered endothelial function. Endothelial dysfunction is the earliest and reversible stage of atherosclerotic disease; therefore, it is an attractive therapeutic target. Moreover, altered endothelial reactivity of the brachial artery reflects that seen in coronary arteries [1]. Therefore, a more aggressive CV evaluation strategy should be considered—serum markers and non-invasive evaluation of endothelial function may be able to identify subclinical atherosclerosis and high-risk asymptomatic subjects with the aim of addressing appropriate lifestyle and pharmacological interventions to minimize the risk.

## 5. Conclusions

This study demonstrates that patients with pSS without clinically evident cardiovascular disease or presence of concomitant cardiovascular risk factors have an altered endothelial function, which indicates a higher susceptibility to the development of atherosclerosis. Furthermore, revealed subclinical CV involvement is directly related to elevated inflammatory injury, postulating that inflammation and disease activity are CVD risk factors in patients with pSS. Since there are no studies investigating the effect of treatment on the incidence of CV events in pSS, further research is needed to explore whether interventions that potentially reduce inflammatory burden could also improve endothelial function in these patients.

## Figures and Tables

**Table 1:** Detailed characteristics of the pSS group.

Parameter (Mean ± SD or *n* (%))	pSS Group *n* = 46
disease duration (years)	5.47 ± 0.68
RF positivity	34 (73.9)
ANA > 1:320	46 (100)
anti-Ro/SS-A antibodies positivity ^	37 (84)
anti-La/SS-B antibodies positivity ^	30 (68.1)
positive labial salivary gland biopsy	42 (91.3)
hypergammaglobulinemia	16 (34.7)
low complement ^#^	6 (13)
leukopenia	15 (32.6)
ESSDAI	9.50 ± 0.86
ESSPRI	4.37 ± 0.29
Anti-malarial drugs use	24 (52.17)
DMARDs use	35 (76)
xerostomia	39 (84.7)
xerophthalmia	39 (84.7)
arthritis/arthralgia *	43 (93.4)
pulmonary involvement * (ILD confirmed by HRCT)	10 (21.7)
parotid enlargement *	8 (17.3)
Raynaud’s phenomenon	5 (10.8)
Lymphadenopathy *	11 (23.9)
renal involvement *	3 (6.5)
peripheral nervous system involvement *	3 (6.5)
cutaneus involvement *	6 (13)

Abbreviations: ANA—anti-nuclear antibody, DMARDs- disease-modifying antirheumatic drugs, ESSDAI—EULAR Sjogren’s Syndrome Disease Activity Index, ESSPRI—EULAR Sjogren’s Syndrome Patient Reported Index, HRCT—high-resolution computed tomography, ILD—interstitial lung disease, RF—rheumatoid factor. * defined by ESSDAI domains, ^#^ reduced C3 and/or C4 levels, ^ evaluated using commercial ELISA kits.

**Table 2:** Demographic and clinical characteristics of entire cohort.

Parameter (Mean ± SD or *n*)	pSS Group *n* = 46	Control Group *n* = 30	*p*-Value
Sex (F/M)	43/3	30/0	NS
Age (years)	47.34 ± 11.9	45.20 ± 11.8	NS
SBP (mmhg)	115 ± 15.59	103.33 ± 13.47	0.0012
DBP (mmhg)	70.43 ± 9.87	64.33 ± 9.35	0.008
BMI (kg/m^2^)	23.52 ± 3.36	23.44 ± 3.6	NS
Physical activity (y/*n*)	23/23	17/13	NS
Glucose (mg/dL)	90.45 ± 10.18	92.6 ± 6.96	NS
Uric acid (mg/dL)	4.8 ± 1.12	4.93 ± 0.94	NS
Total cholesterol (mg/dL)	207.67 ± 35.58	217.7 ± 48.83	NS
HDL (mg/dL)	67.19 ± 16.79	62.86 ± 12.28	NS
LDL (mg/dL)	124.23 ± 30.05	136.16 ± 41.26	NS
Tg (mg/dL)	81.78 ± 35.03	93.26 ± 58.82	NS
Fe (µg/dL)	81.02 ± 25.61	109.84 ± 37.29	0.0001
UIBC (µg/dL)	265.91 ± 60.85	251.5 ± 59.71	NS
TIBC (µg/dL)	346.93 ± 48.33	364.4 ± 46.98	NS
Transferrin (g/L)	2.65 ± 0.41	2.78 ± 0.39	NS
Ferritin (µg/L)	84.21 ± 91.43	59.83 ± 40.54	NS
25-OH-vitamin D (ng/mL)	30.44 ± 9.81	27.47 ± 12.35	NS
BNP (pg/mL)	33.62 ± 26.95	28.58 ± 16.41	NS
Homocysteine (umol/L)	11.05 ± 3.6	9.34 ± 2.14	0.02
Folic acid (ng/mL)	8.97 ± 3.26	10.58 ± 3.24	0.03
Vitamin B12 (pg/mL)	430.39 ± 177.69	415.92 ± 151.68	NS
Waist-hip ratio (WHR)	0.78 ± 0.06	0.81 ± 0.05	0.03
Score (%)	1.26 ± 2.02	0.8 ± 1.37	NS

Abbreviations: BMI—body mass index, BNP—brain natriuretic peptide, SBP—systolic blood pressure, DBP—diastolic blood pressure, Fe—iron, HDL—high-density lipoprotein, LDL—low-density lipoprotein, SCORE—Systematic Coronary Risk Evaluation, TIBC—total iron binding capacity, Tg—triglycerides, UIBC—unsaturated iron binding capacity, SD—standard deviation, NS—not significant.

**Table 3:** Disease-specific variables associated with FMD and biomarkers levels.

Parameter	Univariate Analysis	Multivariate Analysis
FMD	Ro/SS-A antibodies (*r* = 0.34, *p* = 0.03) pulmonary involvement (*r* = 0.52, *p* = 0.001) ESSPRI (*r* = −0.40, *p* = 0.01) ADMA (*r* = −0.35, *p* = 0.05) VEGF (*r* = −0.44, *p* = 0.006) L-arginine (*r* = 0.35, *p* = 0.03)	Ro/SS-A antibodies (*β* = 0.3, *p* = 0.04) pulmonary involvement (*β* = 0.45, *p* = 0.003)
ADMA	ESSDAI (*r* = 0.33, *p* = 0.02) SCORE (*r* = 0.57, *p* = 0.00003) focus score (*r* = 0.38, *p* = 0.04)	ESSDAI (*β* = 0.24, *p* = 0.04)
cGMP	disease duration (*r* = −0.31, *p* = 0.03)	
VEGF	hypocomplementaemia (*r* = 0.40, *p* = 0.005)	
angiostatin	RF (*r* = 0.53, *p* = 0.0001) ESR (*r* = 0.64, *p* = 0.000001) Ro/SS-A antibodies (*r* = −0.31, *p* = 0.03) hypergammaglobulinemia (*r* = −0.51, *p* = 0.0002)	

Abbreviations: ADMA—asymmetric dimethylarginine, cGMP—cyclic guanosine monophosphate, ESR—erythrocyte sedimentation rate, ESSDAI—EULAR Sjogren’s Syndrome Disease Activity Index, ESSPRI—EULAR Sjogren’s Syndrome Patient Reported Index, FMD—flow mediated dilation, RF—rheumatoid factor, SCORE—Systematic Coronary Risk Evaluation, VEGF—vascular endothelial growth factor.

## Data Availability

The data are available from the corresponding author upon request.
